# Computational models of amorphous ice for accurate simulation of cryo-EM images of biological samples

**DOI:** 10.1016/j.ultramic.2023.113882

**Published:** 2024-02

**Authors:** James M. Parkhurst, Anna Cavalleri, Maud Dumoux, Mark Basham, Daniel Clare, C. Alistair Siebert, Gwyndaf Evans, James H. Naismith, Angus Kirkland, Jonathan W. Essex

**Affiliations:** a Rosalind Franklin Institute, Harwell Science and Innovation Campus, Didcot, OX11 0FA, United Kingdom; b Diamond Light Source, Harwell Science and Innovation Campus, Didcot, OX11 0DE, United Kingdom; c School of Chemistry, University of Southampton, Highfield, Southampton, SO17 1BJ, United Kingdom; d Division of Structural Biology, University of Oxford, Roosevelt Drive, Oxford, OX3 7BN, United Kingdom; e Department of Materials, University of Oxford, Parks Road, Oxford, OX1 3PH, United Kingdom

**Keywords:** Electron microscopy, Amorphous ice, Molecular dynamics, Multislice simulation

## Abstract

Simulations of cryo-electron microscopy (cryo-EM) images of biological samples can be used to produce test datasets to support the development of instrumentation, methods, and software, as well as to assess data acquisition and analysis strategies. To be useful, these simulations need to be based on physically realistic models which include large volumes of amorphous ice. The gold standard model for EM image simulation is a physical atom-based ice model produced using molecular dynamics simulations. Although practical for small sample volumes; for simulation of cryo-EM data from large sample volumes, this can be too computationally expensive. We have evaluated a Gaussian Random Field (GRF) ice model which is shown to be more computationally efficient for large sample volumes. The simulated EM images are compared with the gold standard atom-based ice model approach and shown to be directly comparable. Comparison with experimentally acquired data shows the Gaussian random field ice model produces realistic simulations. The software required has been implemented in the Parakeet software package and the underlying atomic models are available online for use by the wider community.

## Highlights

• Molecular dynamics simulations including the largest model of a protein in ice.
• A gaussian random field approach to modelling the amorphous ice potential.
• The gaussian random field and molecular dynamics models results in identical images.
• Simulations with the gaussian random field model are two orders of magnitude faster.

## 1 Introduction

In cryo-electron microscopy (cryo-EM), projection images of samples embedded in amorphous ice are acquired using a transmission electron microscope (TEM). The physics of the electron scattering, and image formation are well understood, and realistic EM images are routinely simulated using the multislice algorithm [1], [2]. These simulations can assist in the interpretation of experimentally acquired cryo-EM images. They also allow the limitations [3] of different data acquisition schemes to be evaluated by performing *in silico* experiments that sample the available space of data acquisition parameters [4]. TEM simulations are a key component in the creation of a “digital twin” that models a real microscope, sample, and detector. Such a digital twin can be used to determine optimal data acquisition schemes, design new hardware, and provide phantom test datasets that can be used in the development of data processing and analysis software.

Ice exists in several crystalline and amorphous forms; the most common being hexagonal crystalline ice [5]. As well as reducing image quality as a result of diffraction, crystalline ice often causes severe damage to most biological structures by withdrawing water molecules from the hydration shells of the specimen or from the specimen itself [6,7]. In contrast, amorphous ice preserves the specimen in a near native state, avoiding the formation of ice crystals, and the formation of amorphous ice drives most cryo-EM protocols [6]. Amorphous ice does not have the long-range order present in crystalline ice but instead retains some of the structural characteristics of liquid water, although there is some indication that structural differences exist between amorphous ice and structurally arrested liquid water [1]. Amorphous ice is found in two main forms, low-density amorphous (LDA) ice, the form present in cryo-EM samples, with a density around 0.94 g/cm^3^ [5], [6], [8], [9], [10], [11], a value lower than the density of liquid water but higher than that of hexagonal crystalline ice, and high-density amorphous (HDA) ice with a density greater than 1.00 g/cm^3^ that can only be formed under high pressures [5].

To avoid ice crystallisation, specimens must be cooled rapidly, with typical cooling rates higher than 10^5^ Ks^−1^ [12]. This cannot be achieved using liquid nitrogen due to the Leidenfrost effect [13,14]; instead, to obtain amorphous ice, thin samples (typically less than < 10 µm) are prepared by rapidly plunging the sample into a reservoir of liquid ethane [6]. For specimens such as cells, tissue sections, or entire organisms that are too thick to be plunge frozen without crystalline ice formation, high-pressure freezing has been shown to be effective up to specimen thicknesses of around 200 µm [7], [12], [15].

Consequently, simulation of any cryo-EM experiment, especially cryo electron tomography (cryo-ET) data from thick samples of cells, must account for substantial volumes of amorphous ice [6]. The density and thickness of the ice plays a significant role in the simulated image contrast [16]. The multislice algorithm [2,17] starts from an atomic model which is then divided into thin slices along the direction of the incident electron beam such that each slice can be considered as a weak phase object. The atomic potential in each slice is typically calculated as a sum of the contributions from the individual atoms in the sample; the electron wave is transmitted through each slice and Fresnel propagated to the next slice to produce a complex sample exit plane wave which is then multiplied by the microscope contrast transfer function (CTF) in Fourier space and squared in real space to produce a simulated phase contrast TEM image [16,18]. The gold standard method to produce a physically realistic ice model for the simulation of EM images is to place the required biomolecule(s) in a volume of water and then to perform a relaxation using molecular dynamics to produce a model that places the individual water molecules in physically realistic positions [16].

However, to produce useful phantom datasets for tomography, large sample volumes are required with a correspondingly large number of water molecules. For example, a biological sample embedded in a planar lamella of LDA ice with dimensions of 400 × 400 × 150 nm would contain more than 750 million water molecules. Performing molecular dynamics simulations of water molecules using a cubic sample volume of side length 645 Å containing 26.5 million atoms took over 18 h on the ARCHER supercomputer [19] using 24 high-memory nodes, each containing 24 cores. Expanding the size of the simulation box even further to be consistent with a typical biological sample used in cryo-EM, and then performing molecular dynamics simulations would be impractical. Moreover, simulation of a single TEM image from a 400 × 400 × 150 nm lamella required more than a day on a Nvidia Quadro P4000 GPU. This demonstrates that the simulations required for a tomographic tilt series rapidly become computationally too expensive. Therefore, whilst a combination of physically realistic atom-based models and the mutlislice algorithm can be used for small volumes, computationally simpler methods are required for larger sample volumes that are the norm in cryo-ET. Naturally, these new methods must give results consistent with both the established gold standard and experimental data.

We have used molecular dynamics software to produce atomic models of water with and without an embedded apoferritin particle as our ‘gold standard,’ under the assumption that, for the purpose of TEM image simulation, LDA ice can be modelled as structurally arrested water. We also evaluated a more computationally efficient Gaussian Random Field (GRF) continuum approach to model the atomic potential of the amorphous ice. We show that both methods produce equivalent simulated EM images, and we have further validated the simulations against an experimental dataset. The GRF model is implemented within the open-source Parakeet digital twin software package [4], which uses the MULTEM simulation library internally [18]. The atomic coordinates of the physical atom-based model are available for download from Zenodo [20] to enable other researchers to make use of them in future experiments.

## 2 Methods and materials

### 2.1 Atomic model of apoferritin

**Fig. 1:**
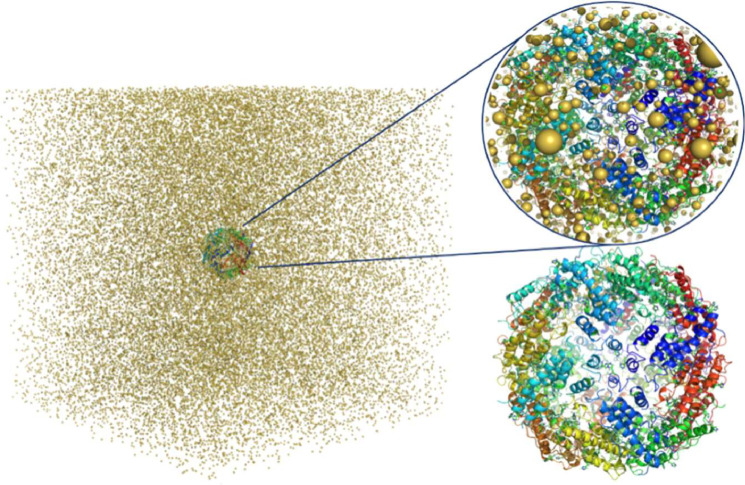
Apoferritin model embedded in water. Note that to illustrate the size of the simulation volume relative to the protein environment, water molecules are excluded from the visualisation and only the free ions in the model (e.g., sodium) are shown. The apoferritin molecule (shown zoomed in the inset image) is at the centre of the simulation volume.

The atomic coordinates of apoferritin, entry 6Z6U [21] available from the Protein Data Bank (PDB) [22] were used. The structure is resolved to 1.25 Å and was determined by single particle cryo-EM. The model has a total structural weight of 511.09 kDa, and 38,846 atoms in 4152 residues; bound waters were removed from the model. Apoferritin is commonly used as a test sample for cryo-EM applications [23] since it has a high degree of symmetry which aids reconstructions along different axes. Alpha helices are also easily identified from low resolution maps which aids in a qualitative assessment of the reconstruction quality. A model of apoferritin in water is shown in Fig. 1.

### 2.2 Molecular dynamics simulations

We chose to create a system that was sufficiently large to resemble the depth of amorphous ice typically present in cryo-EM samples. The system was constructed as a single large cube to minimise diffraction effects from the edges of the cube. The size of the “ocean” model reported by Lagardère et al. [24] provides a water box of size 615^3^ Å^3^, which served as a benchmark for this work. Experimental datasets will have a higher density of protein molecules which will alter the positions of the water molecules, but such changes would not be discernible and thus unlikely to be relevant to the simulation.

The simulations were run using the Iridis5 HPC cluster, the ARCHER supercomputer, and the ARCHER2 supercomputer. The Visual Molecular Dynamics program (VMD) [25] was used for system setup and the molecular dynamics (MD) simulations were performed using NAMD version 2.13 [26] with the CHARMM36 forcefield [27]. Details of the CPU resources required to run the simulations are provided in Table 1. It is important to note that in the case of the ARCHER2 simulations, the individual nodes were run at half-capacity to mitigate issues with overloading the RAM.

**Table 1:** System dimensions of the models used in the molecular dynamics simulations with the corresponding number of atoms contained in each system, the number of cores used in each case, and the computing cluster used to run the simulations. *The 723^3^ Å^3^ system includes the apoferritin molecule and counter ions. All other systems contain TIP3P water only.

Dimensions (Å^3^)	No. atoms	Compute Cluster	Resources (equiv. no. cores)
81 × 81 × 81	50,892	Iridis 5	40
243 × 243 × 243	1,408,797	Iridis 5	120
486 × 486 × 486	11,323,362	ARCHER	576
567 × 567 × 567	18,004,077	ARCHER	576
645 × 645 × 645	26,520,918	ARCHER	576
723 × 723 × 723 *	38,844,502	ARCHER2	12,800
735 × 735 × 735	38,997,612	ARCHER2	12,800

The sizes of the systems studied here ranged from a box size of 81^3^ Å^3^ containing 50,892 atoms, to a 735^3^ Å^3^ box containing over 38 million atoms. The number of atoms in each system is shown in Table 1. The Langevin thermostat with a damping coefficient of 1 ps^−1^ was used for NVT (constant number of particles, constant volume, and constant temperature) equilibration [28], and the Nose-Hoover Langevin-Piston barostat with reference pressure 1 atm was used for NPT equilibration (constant number of particles, constant pressure, constant temperature) and production [29,30]. Every system employed periodic boundary conditions and the Particle Mesh Ewald (PME) method [31] with default grid spacing of 1 Å was used to account for long range electrostatic forces. The CHARMM TIP3P water model [32] was used to add explicit solvent through the Solvate plugin, and bonds to hydrogen were kept rigid using the SHAKE algorithm [33]. For the initial simulations of bulk water, a 2 fs timestep was used. The default conjugate gradient algorithm was used for energy minimization, and equilibration was performed for 25,000 steps under NVT and then NPT conditions. Production MD was performed under NPT conditions for 1 million steps for the smaller systems (up to 243^3^ Å^3^). This was subsequently decreased to 100,000 steps as the size of the systems increased to limit computational expense.

For the systems containing apoferritin, psfgen was used to add the appropriate hydrogens to the crystal structure to model physiological pH, and the Autoionize plugin was employed to model neutrality at 0.15 M with Na^+^ and Cl^−^ ions. The 6Z6U crystal structure contained 24 cysteine residues which had oxidised to s-oxycysteine [21]. For the purposes of this work, the inclusion of these modified cysteines in the protein structure was deemed to be negligible, and the setup used regular cysteine residues in place of the s-oxycysteine. For construction of the large solvated apoferritin model, the MergeStructs plugin was used to assemble the input coordinates. The simulations of apoferritin were run with an unconstrained protein to capture any protein dynamics. After the final simulation (Table S2), position restraints were applied to the protein to restrain to the crystal positions, and analysis was performed using these coordinates.

To fully equilibrate and run a system of this size required extensive simulation time and was not without complication. In addition to the protocol outlined here, further details about the simulations can be found in the Supplementary Information (Table S2). The thermostat and barostat used were the same as for the bulk water simulations. Firstly, a gradual heating protocol was employed, under NPT conditions, at 50 K, 100 K, 150 K, 220 K, 250 K. Each of these simulations was run with 20,000 minimisation steps and 80,000 production steps using a 2 fs timestep, equating to a total simulation time of 1 ns at this stage. After reaching 280 K, the system conditions were altered to NVT. After one simulation at 280 K, the timestep was lowered to 1 fs. The temperature was raised to 298 K, where the system was minimised for 50,000 steps and production was run for 100,000 steps (Table S2, simulation 7). To adjust for the density of liquid water, the dimensions of the box were gradually increased over a further 5 concurrent NVT simulations using this adjusted protocol, followed by a series of short simulations under NPT conditions to allow the pressure to equilibrate. The final coordinates were obtained at 298 K, with the density of water being 1.009 g cm^−3^, with box dimensions of 723 × 723 × 723 Å. The total amount of simulation time was 2.11 ns. To our knowledge, this is the largest solvated model of a biological macromolecule publicly available. Importantly, the size of the simulation box means that it can be usefully applied to the analysis of simulated cryo-EM images. Analyses were performed using the MDAnalysis Python package [34,35]. These coordinates are supplied via Zenodo [20] with associated scripts provided on GitHub [36].

### 2.3 Simulation of EM images

To simulate TEM images, Parakeet [4], a simulation package based on the MULTEM library [18] was used. MULTEM provides a GPU accelerated implementation of the multislice algorithm [2,17] and a model of the microscope optics. These algorithms were extended and wrapped using the Pybind11 C++/Python binding package [37] to create a simple Python API. The software is open source and can be obtained from the Rosalind Franklin Institute GitHub repository.

The multislice algorithm is used to analytically solve the Schrödinger equation for the elastic interaction between matter and electrons, incorporating multiple scattering of electrons through the sample [2,17]. The implementation used here takes an atomic model of the sample and then calculates the total atomic potential as the sum of the independent atomic potentials of the constituent atoms in the model. The atomic potential is then divided into slices of a given thickness along the direction of the electron beam [16,18]. The thickness of each slice is typically such that each slice can be considered as a weak phase object; in the simulations used here, the slice thickness is 5 Å. The atomic potential in each slice is then projected onto an infinitesimally thin plane and the atomic potential between the planes is considered to be zero. The wave function, $\psi_{n}\left(x,y\right)$, is transmitted through the slice by multiplying by the transmission function, $t_{n}\left(x,y\right)=\exp\left(i\sigma \int_{z_{n}}^{z_{n}+\Delta z}V\left(x,y,z\right)dz\right)$, where z_n_ is the depth of the slice, Δz is the slice thickness, V(x,y,z) is the three-dimensional atomic potential and σ is the interaction parameter [38]. The wave function is then propagated to the next slice by convolving with the Fourier transform of the Fresnel propagator, $P_{n}\left(k,\Delta z\right)=\exp\left(-i\pi \lambda k^{2}\Delta z\right)$, where λ is the electron wavelength. The wave function at slice *n* + 1 is then given by $\psi_{n+1}\left(x,y\right)=F^{-1}\left\{P_{n}\left(k,\Delta z\right)\times F\left[t_{n}\left(x,y\right)\times \psi_{n}\left(x,y\right)\right]\right\}$ [38]. This process is continued for each slice in the sample until the electron wave is propagated through the whole sample resulting in a complex wave function at the exit plane of the final slice of the sample. In this work the effects of the microscope optics were modelled by the application of a phase contrast transfer function (CTF). This is an oscillating phase shifting complex function applied in Fourier space and is determined by the microscope aberrations which apply a phase shift to the exit wave. The CTF is dampened at high resolution due to the effects of partial spatial and temporal coherence which were modelled here as simple multiplicative envelopes which is appropriate for the case of a weak phase object [38,39].

### 2.4 Statistical model for amorphous ice

To calculate accurate EM image simulations of biological specimens embedded in amorphous ice, a physical model of the ice that has been relaxed using molecular dynamics is most accurate [16]. However, as the volume of the sample increases, this type of physical model for the amorphous ice becomes impractical to compute. Therefore, a much more computationally efficient approach is required for samples with large volumes of amorphous ice.

**Fig. 2:**
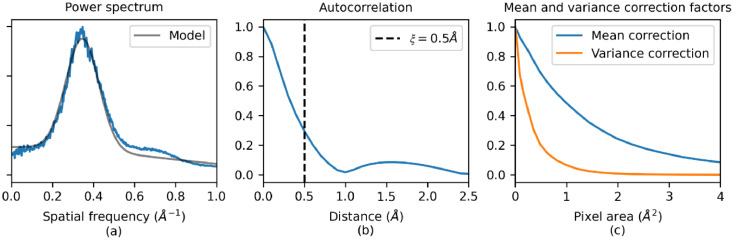
Power spectra and fitted Gaussian functions (a); autocorrelation function with the effective correlation length, ξ, given by the vertical dashed line (b); the mean and variance correction factors given by the ratio of the observed to expected mean potential and variance of the potential respectively (c).

Observations of thin films of amorphous carbon have shown that, for amorphous materials above a certain thickness of around 10 nm [40], EM images of different samples are qualitatively indistinguishable, and high-resolution structural details within the specimen cannot be interpreted [41]. Therefore, we reason that using a continuum approach to model amorphous ice rather than an atomistic model may not result in any loss of distinguishable information in the simulated EM images. A typical continuum model where the amorphous ice potential is modelled at every position in space by a mean potential is simple but gives a single value for the potential resulting in a featureless image of the ice. The amorphous ice itself contributes to some features in the simulated EM images which can be suitably modelled by Fourier filtered noise [42]. Therefore, a reasonable approach is to model the atomic potential of the amorphous ice as a GRF with a given mean, variance, and power spectrum such that the GRF potential has the same statistical characteristics as the atomic potential from a physical model in each slice of the multislice calculation. In this model, the correlation length, ξ - defined as the distance at which the autocorrelation function drops to a value of 1/e of its zero-lag value - is assumed to be much smaller than the slice thickness such that, whereas correlations orthogonal to the beam are preserved, those parallel to the electron beam are lost [43]. As shown in Fig. 2b, the value of ξ is ∼0.5 Å so this approximation is appropriate for typical slice thicknesses of 3–5 Å.

The expected value of the distribution of the atomic potential can be calculated from the electron scattering factors, $f_{e}^{Z}$, in Fourier space, where Z is the atomic number. There are various parameterisations for these scattering factors; however, in this paper we use a parameterisation in terms of hydrogenic wavefunctions described by [44]. The mean potential for a single atom is given by $V_{0}=\;\frac{1}{\kappa }f_{e}^{Z}\left(0\right)$, where $\kappa =2\pi \hbar^{2}/m_{e}q_{e}$ with *m_e_* the electron rest mass and *q_e_* the elementary charge constant. The expected potential per pixel with N water molecules per unit area, which is approximately 145.59 N (eV *Å*^−2^), can then be given in terms of the electron scattering factors for oxygen, $f_{e}^{8}\left(0\right)$, and hydrogen, $f_{e}^{1}\left(0\right)$, as follows:

(1) $$V_{0}=\;\frac{N}{\kappa }\left(f_{e}^{8}\left(0\right)+2f_{e}^{1}\left(0\right)\right).$$

The shape of the power spectrum is modelled as a sum of two Gaussian functions: the first is centred at the origin in Fourier space to represent the contribution of the single atom atomic potential function to the power spectrum and has a variance $s_{1}^{2}\;$and a scale factor $a_{1}$; the second is centred at a spatial frequency, $m=\frac{1}{2.88}{Å}^{-1}$, which corresponds to the average distance between the oxygen atoms in LDA ice [16]. The power spectrum at a given spatial frequency, q, is thus:

(2) $$P=a_{1}e^{-q^{2}/\left(2s_{1}^{2}\right)}+a_{2}e^{-{\left(q-m\right)}^{2}/\left(2s_{2}^{2}\right)}.$$

The relative weightings of these Gaussians and their scale parameters were determined by fitting the power spectrum model to the observed power spectrum simulated from the physical ice model as shown in Fig. 2a, giving parameters, a_1_ = 0.199, s_1_ = 0.731, a_2_ = 0.801, s_2_ = 0.081. Finally, the variance scales linearly with the number of water molecules per unit area such that $\sigma_{N}^{2}=\sigma_{0}^{2}N=\;10,195.82N\left({Å}^{-2}\right)$.

In practice, in the MULTEM software, the atomic potential for each atom is calculated once for each element in a finite grid; as the pixel size increases, the sampling of the atomic potential of the atoms decreases. Furthermore, the potential function is interpolated and truncated at small distances from the atom position to avoid an infinite potential at zero distance. These two implementation details result in the overall sum of the atomic potential values on the potential grid tending to be smaller than the expected sum, with the difference increasing as the size of the pixel increases; likewise, the variance will also tend to decrease. Therefore, a correction factor needs to be applied to both the mean and the variance based on the size of the pixels. Fig. 2c shows these correction factors; the ratio of the observed to expected mean and variance as a function of pixel area. For the GRF model, for a given pixel area, the correction factor was determined by using a lookup table to interpolate from the pre-computed values. The elements in these lookup tables are given in Table S1 in the supplementary material.

Given a mean, variance, and power spectrum, the GRF model is generated in Fourier space by using the power spectrum to compute the amplitudes of the amorphous potential and then assigning uniformly distributed random phases. Taking the inverse Fourier transform then gives the GRF which can be normalised to the desired mean and variance. The GRF is masked to only include pixels where amorphous ice potential is expected, and the amorphous ice potential is then added at every voxel with a protein potential below a given threshold. Increasing the threshold allows the amorphous ice potential to overlap with the atomic potential of the protein, whilst reducing the threshold to zero eliminates overlap. Allowing the amorphous ice potential to overlap with the protein potential increases the phase shift at these positions, increasing the contrast. A threshold value of V_0_ / 3 was chosen to give good agreement between the image contrast produced by the physical and random models. A beneficial side effect of this method is that each calculation produces two GRFs with the same characteristics, one in the real and one in the imaginary component of the transformed image. Therefore, this procedure only needs to be performed once for every two slices in the multislice calculation.

### 2.5 Tomographic reconstruction and analysis

The oscillation of the CTF results in contrast inversions at spatial frequencies where the CTF is negative. The location of the first zero crossing in the CTF defines the Scherzer point resolution [45]; which is the maximum directly interpretable resolution obtainable without any additional correction. To obtain higher resolution reconstructions, CTF correction, which corrects the real valued image intensities [46,47], or exit wave reconstruction, which yields the complex valued exit wave [48], must be performed. Additionally, for thick samples, different voxels within a sample will have different defocus values. In this case, CTF correction using a single defocus value will not be sufficient and a 3D CTF correction is required [49]. Therefore, in this analysis, in order to reconstruct the tomographic tilt series data, 3D CTF correction [50] with the weighted back projection (WBP) algorithm [51], [52], [53] is performed using a GPU accelerated filtered back projection (FBP) algorithm from the Astra toolbox [54] through the Tomopy python package [55,56] as implemented in the Parakeet digital twin software package [4].

Since the images are simulated from a known atomic model in this analysis, it is straight forward to fit the original atomic model back into the reconstructed map. To do this, Chimera [57] was used to dock the atomic model into the reconstructions and the fit was further refined with the use of REFMAC5 [58] with rigid body restraints. REFMAC5 produces statistics describing the quality of the fit of the model to the reconstructed map and provides a convenient method for obtaining objective measures of the quality of reconstruction with different parameters. The metric used in this analysis to assess the quality of the reconstruction with the known model is the Fourier shell correlation (FSC) average defined as [59,60]:

(3) $$FSC_{average}=\frac{\sum_{i=1}^{N_{shell}}N_{i}FSC_{i}}{\sum_{i=1}^{N_{shell}}N_{i}}$$

Where N_i_ is the number of elements in a shell and FSC_i_ is the FSC within that shell.

### 2.6 Experimentally acquired apoferritin dataset

Simulated images should be realistic representations of experimental data collected. Accordingly, simulated images of apoferritin were validated against an experimentally collected apoferritin dataset. The experimental dataset was collected on a Krios 2 microscope at eBIC using a K3 detector with correlated double sampling at 300 keV using apoferritin mounted on Carbon grids. The thickness of the amorphous ice covering the sample was estimated by cryo-ET to be approximately 20 nm. The tilt range used was ± 60° with a tilt angle of 3° between each tilt position. At each tilt position a movie with 10 frames was acquired and summed to produce an image. The effective pixel size at the sample was 1.34 Å and the total electron dose over the whole dataset was 100 e^-^/Å^2^ giving a dose of 2.5 e^-^/Å^2^ per image or 4.49 e^-^/pixel. A defocus of −2.5 µm was used.

## 3 Results

### 3.1 Ice models

The TIP3P water model [61] is one of the most well-recognised water models employed in molecular dynamics simulations of biological macromolecules. Typically, these systems contain thousands of explicit water molecules; several orders of magnitude smaller than the number of water molecules reported here (Table 1). To assess the robustness of the physical model, the trajectories of the MD simulations were inspected for energy and density drift. The density of the water was calculated as:

(4) $$\rho_{\mathrm{water}}=\frac{m}{V}=\frac{Mr_{\mathrm{water}}N_{\mathrm{water}}}{N_{A}\;V}$$

Where *m* is the mass of the system in grams, *V* is the volume in cm^3^, *Mr_water_* is the molar mass of water (18.015 g mol ^−1^), *N_water_* is the number of explicit water molecules in the system and *N_A_* is Avogadro's number. All systems showed consistent energy and density drift at 298 K. TIP3P water properties give a density of 0.982 g cm^−3^, compared to the experimental density of liquid water reported by the same authors as 0.997 [61]. For simulation systems on the order of thousands rather than millions of water molecules, this density is consistent under NPT conditions, as demonstrated by Samways [62]. The densities in this work are consistently reported as close to 1 g cm^−3^.

The O—O radial distribution function (RDF) for the 645^3^ Å^3^ system was calculated to assess the structure of the liquid water model at 298 K. To reduce the computational expense, the radial distribution was calculated for the final frame of the 645^3^ Å^3^ water system, as this was the smallest box that was representative of a homogenous fluid and assessing the radial distribution for this box was assumed to be representative of bigger systems. The fluid structure confirmed that the TIP3P water model produces a physically realistic model of the water for a box of this size. The appropriate RDF is included in the Supplementary Information (Fig. S2).

### 3.2 Validation of the statistical properties of the ice models

**Fig. 3:**
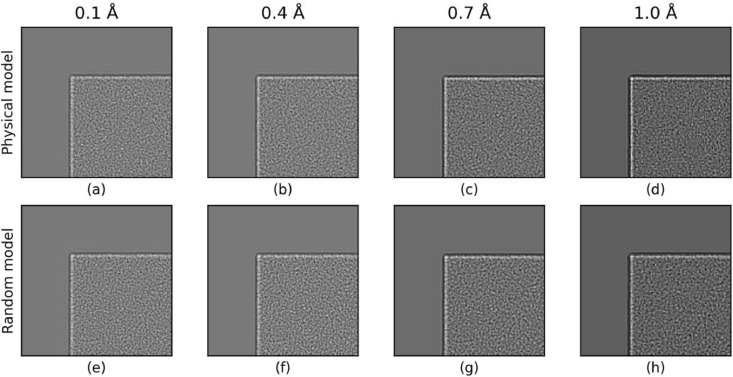
Squared amplitude of the simulated exit waves from the physical atom-based model (a-d) and the GRF model (e-h) showing an edge of the cubic sample for different pixel sizes. The noise structure and the edge effects seen in the ice image using the physical model are qualitatively preserved in the GRF model.

Images were simulated from the 723^3^ Å^3^ MD water model containing 38,844,502 water molecules with a slice thickness of 5 Å and with a range of grid sampling sizes from 0.1 Å to 1.0 Å. Equivalent images were then also simulated using the GRF model. Table S3 in the supplementary material shows the full list of simulation parameters used. Simulations with the amorphous ice potential using the GRF model require much less computational time than with the physical model; TEM images from the physical model and GRF model were simulated in 693 s and 63 s respectively on a Nvidia Quadro P4000 GPU. The simulated exit waves were then evaluated to determine whether simulations from the two models were distinguishable from one another. Fig. 3 shows the squared amplitude of the simulated exit waves from the physical and GRF models, highlighting a corner of the cubic sample. The noise structure in the images and the edge effects seen at the interface between the ice and the vacuum are qualitatively similar. However, since the GRF model uses a sharp-edged mask to define regions containing amorphous ice potential, whereas in the physical model there is some variation in the positions of the atoms along the boundaries of the model, the edge in the physical model image is slightly more diffuse.

**Fig. 4:**
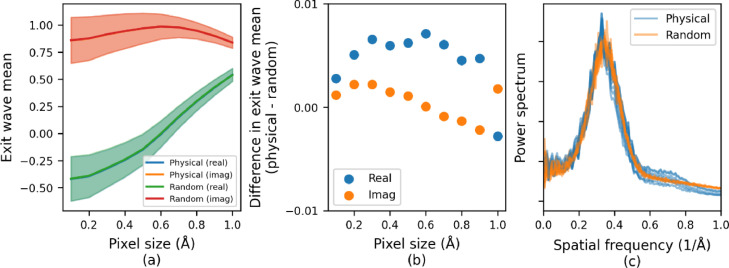
(a) Mean real and imaginary components of the complex exit wave for the physical atom-based model and the GRF model as a function of pixel size. In each case, the distributions of pixel values are comparable for the two models and the continuous errors bars (the shaded areas in the plot) show the standard deviation. It should be noted that, in the plot, the blue and green lines, corresponding to the real components for the physical and random models respectively, overlap considerably as the difference in the values is small. The same is true for the orange and red lines, corresponding to the imaginary components for the physical and random models, respectively. (b) The difference between the real and imaginary components of the exit wave means from the physical and random models as a function of pixel size. (c) Power spectra of the exit waves for the physical atom-based model and the GRF model. The power spectra have peaks at the same spatial frequency and drop off at high and low spatial frequencies at the same rate. The power spectra are plotted for a range of pixel sizes between 0.1 Å and 1.0 Å and can be seen to be comparable.

To perform a quantitative comparison of the simulated exit waves obtained from the atom based and GRF water models, distributions of the real and imaginary components of the pixel values in the exit waves from the interior of the two simulated images were analysed. Fig. 4a shows the mean and standard deviation of the exit wave pixel values for the real and imaginary components for both models plotted as a function of grid sampling pixel size with the standard deviation and the continuous error bars shown in the figure. The distributions of pixel values in both the real and imaginary components are consistent across all grid sampling sizes with exactly the same mean and standard deviation as a function of pixel size. This is further demonstrated in Fig. 4b which shows the difference between the real and imaginary components of the exit wave means from the physical and random models.

Finally, the power spectra calculated from exit waves for the physical atom-based model and the GRF model are shown in Fig. 4c. The power spectrum is the Fourier transform of the auto correlation of the image and determines the correlation length of the noise in the exit wave. In each case the power spectrum shows a peak at approximately the same spatial frequency and drops off at the same rate for both high and low spatial frequency limits indicating that the noise structure in the two simulated images is broadly consistent. Since the simulated exit waves from the physical atom-based model and the GRF based model show the same statistical behaviour in terms of their moments and power spectrum, we conclude that the GRF model can be used in place of the physical atom-based model in simulations.

### 3.3 Validation of simulated images with experimental data

**Fig. 5:**
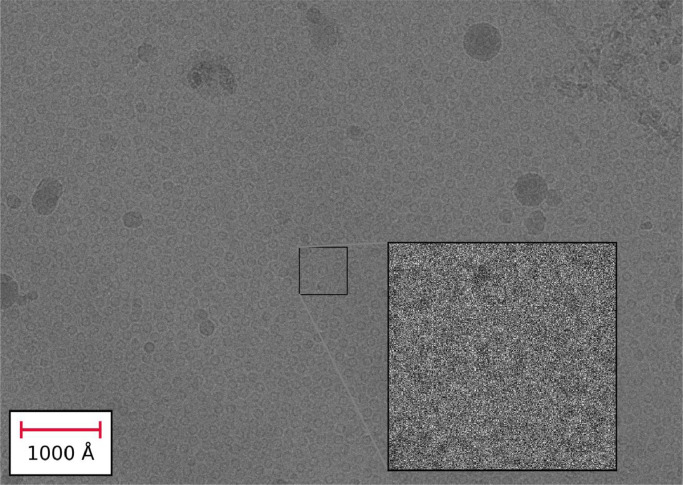
Image from a tomographic data collection containing a large number of apoferritin particles embedded in amorphous ice, which was estimated, using cryo-ET, to have a thickness of approximately 20 nm. The enlarged section shows an image patch, containing a small number of apoferritin particles, used to validate the simulated data.

**Fig. 6:**
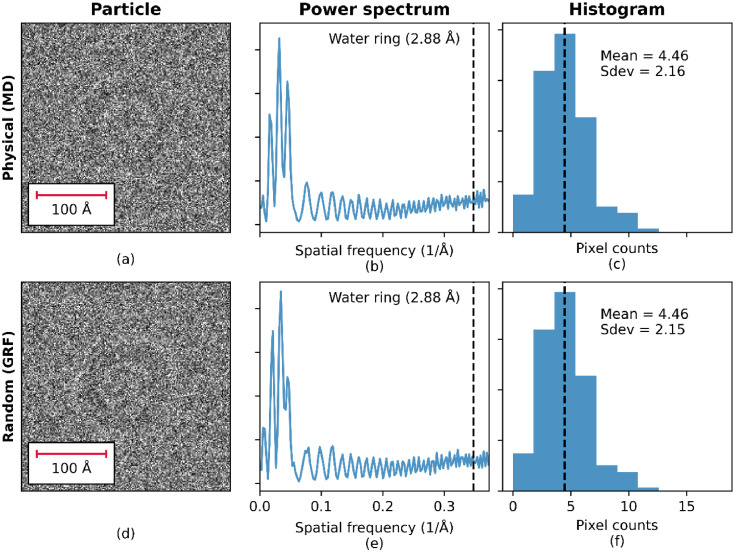
Simulated images using the physical model with MD simulation (a) and GRF model (d). Power spectra calculated from the physical model with MD simulation (b) and GRF model (e). Histograms for the physical model with MD simulation (c) and GRF model (f).

To compare experimental and simulated images, a region containing several apoferritin particles was extracted from an experimental image as shown in Fig. 5. A set of images were then simulated for the same estimated ice thickness, defocus, and number of electrons per pixel. Images were simulated using the two different models to represent the amorphous ice component. In the simulations using the physical (MD) model, the sample was modelled by an atomic model of apoferritin embedded in water which was relaxed using molecular dynamics. Specifically, the model used in this analysis is the 723^3^ Å^3^ water box containing apoferritin described in Table 1. In this model, the amorphous structure of the water and the distances between the water molecules and the atoms of the apoferritin are physically realistic for a particle embedded in water at room temperature. It is assumed that for the purposes of simulating EM images, amorphous ice can be considered as equivalent to structurally arrested water. In this model, the apoferritin particle is in the centre of the ice volume. To obtain a sample 20 nm thick, all atoms outside the central 20 nm z-slice were deleted from the model. In the simulations using the random GRF model, the GRF based ice model was used with the amorphous ice potential simulated as Fourier filtered random noise with a specific power spectrum as described previously. Fig. 6 shows these simulated images with an enlarged apoferritin particle, the power spectrum of the image and the histogram of pixel values. The simulated images from the physical (MD) and random (GRF) models appear qualitatively similar, and their power spectra and histograms also match.

**Fig. 7:**
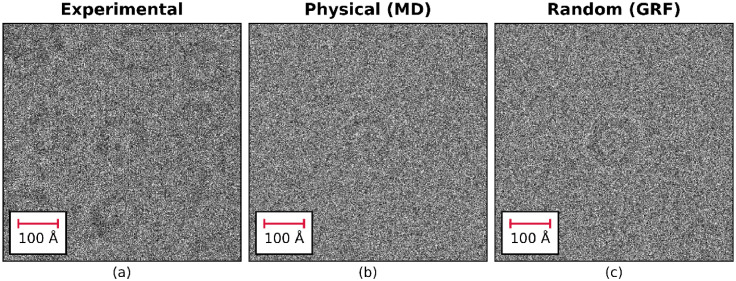
Experimental data containing a large number of apoferritin particles (a) and simulated images using the physical model with MD simulation (b) and GRF model (c) each containing a single apoferritin particle.

Apoferritin particles within a selected region of the experimental image were then identified and a mask delineating the signal and background pixels was generated. Fig. 7 shows the extracted region of the image containing the apoferritin particles, together with the simulated images containing an apoferritin particle in a region with the same field of view. The particles in the simulated images appear to be qualitatively similar to the particles in the experimentally collected image. One difference between the experimental image and the simulated images is that experimental image contains a large number of apoferritin particles closely packed together whereas the apoferritin particles in the simulated images have a large amount of empty space around them containing only density due to the amorphous ice. In terms of the exact position of the atoms in the physical atom-based water model, the presence of additional apoferritin molecules in close proximity will have an effect; however, it is unlikely that these high-resolution structural differences would be visible either experimentally or in the simulated EM images.

To perform a quantitative comparison of the images, the contrast-to-noise ratios of the experimental and simulated images were calculated as:

(5) $$CNR=\frac{\sqrt{\frac{1}{N}\sum_{i=0}^{N}{\left(s_{i}-\mu_{B}\right)}^{2}}}{\sigma_{B}}$$

**Fig. 8:**
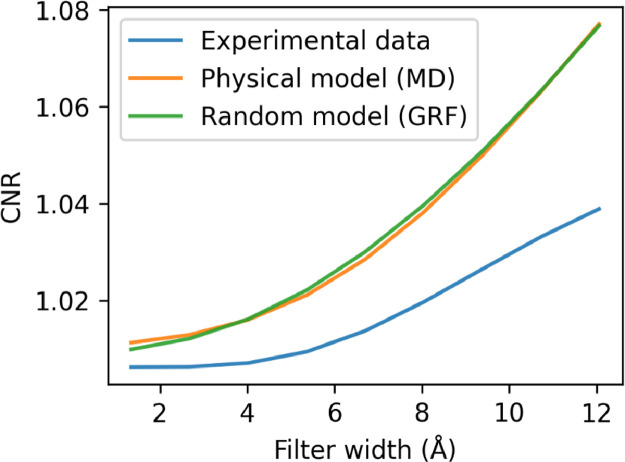
Contrast to noise ratio (CNR) for experimental data (blue), physical (MD) model (orange) and random (GRF) model (green) as a function of low pass filter width.

Here, N is the number of signal pixels, s_i_, µ_B_ is the mean value of the background pixels and σ_B_ is the standard deviation of the intensity in the background pixels. As shown in Fig. 8, the simulated images from both the MD and GRF models have similar contrast, although the simulated images tend to show somewhat higher contrast than the experimental image which may be due to the fact that the true thickness of the ice in the experimental data is not known exactly but estimated from the tomograms.

### 3.4 Validation of reconstructed tomograms from simulated images

**Fig. 9:**
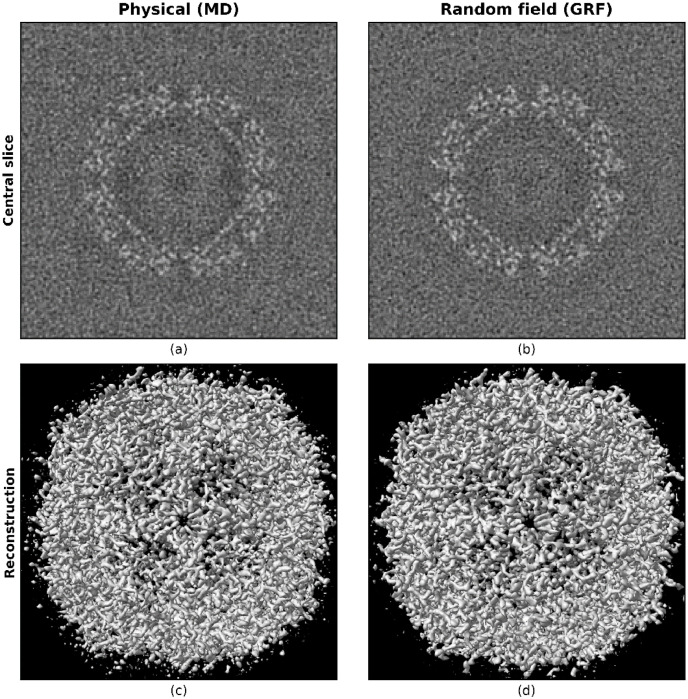
Central slice of a tomographic reconstruction (top) and the 3D reconstruction (bottom) from the physical model with MD simulation (left) and GRF model (right).

**Fig. 10:**
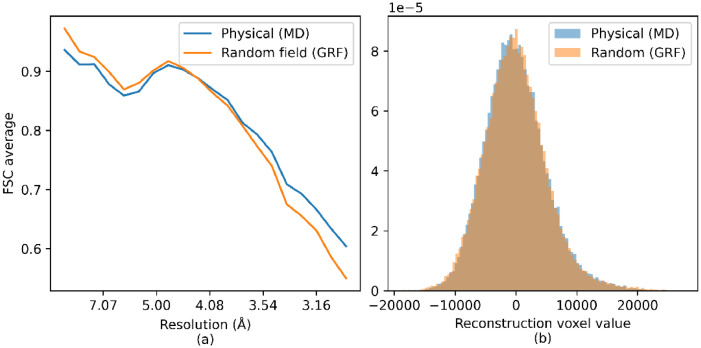
FSC vs resolution between the true model and the reconstructions using 180 images for each of the ice models (a). The histogram of voxel values in the reconstructions from the MD and GRF models (b).

The models used for the amorphous ice described in this paper were further evaluated by tomographic reconstruction using simulated images. To perform this analysis, the physical atom-based MD model was first “shaped” into a cylindrical geometry and a series of projections were calculated over *a* ± 90° tilt range. Equivalent images were then simulated using the GRF model. In a typical cryo-ET experiment, a full 180° rotation is not available due to instrument and sample limitations; however, the purpose of this analysis is solely to ensure that the choice of model for the amorphous ice does not have a significant effect on the quality of the tomographic reconstruction. Although, in general an experimentally collected dataset will have many particles in close proximity and the simulations performed here have a single particle embedded in the volume, the positions of the water molecules around the particles will not be discernible in either the images or the reconstructed tomograms. The central slices and the 3D reconstructions from these two models are shown in Fig. 9 where the reconstructions appear to be qualitatively similar. The distributions of the voxel values in the reconstructed volumes are shown in Fig. 10b and are seen to be quantitatively similar.

For each tilt series, the reconstruction quality was assessed using the FSC average to a resolution of 3 Å between the reconstructed map and the known model. Fig. 10a shows the FSC vs. resolution for the two ice models. The FSC vs. resolution is broadly consistent between the two ice models over the resolution range, although the values for the GRF model appear to be slightly higher at low resolution. Fig. 10b shows the histogram of voxel values in the reconstructed volume. The distributions appear similar and comparison of the two distributions using the Kolmogorov Smirnoff test determined that the null hypothesis where the samples were drawn from the same distribution could not be rejected at the 5 % significance level. The value of the test statistic was 0.009 with a p-value of 0.07. This minor difference may be because in the atomic model, the water atoms are placed at physically realistic distances from the apoferritin atoms whereas in the GRF model the amorphous ice potential is added at every voxel with a potential below a given threshold. Our primary motivation is to enable the meaningful simulation of large volume cryo-ET test datasets to support the development of new data processing and analysis software and to determine optimal data acquisition and processing strategies. For this the ability to produce realistic datasets in a time and computationally efficient manner is crucial, particularly if unsupervised machine learning is to be employed. The reconstruction quality between the atom-based ice model and the GRF model is similar, but the latter is over two orders of magnitude less computationally expensive demonstrating that the GRF model represents a valid tool for such large volume simulations.

### 3.5 Large volume simulation

**Fig. 11:**
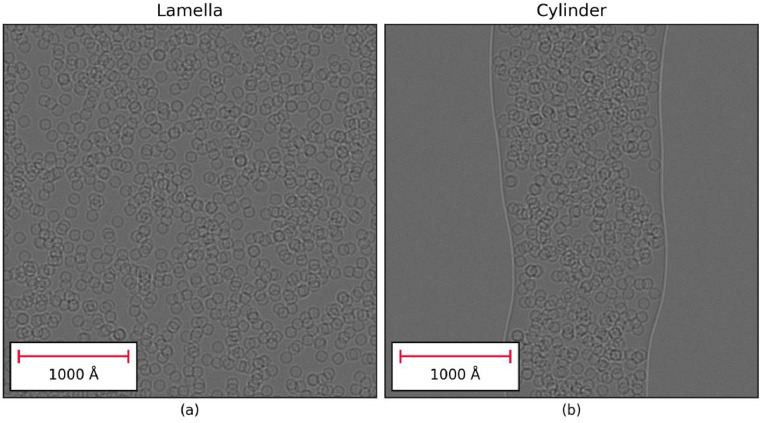
(a) Planar lamella sample of size 400 × 400 × 150 nm containing 1500 apoferritin particles; (b) a cylindrical volume with diameter 150 nm with “natural” edges containing 800 apoferritin particles. These sample volumes contain a large number of water molecules and would not be practical to simulate using an atom-based model. However, using the GRF model full tilt series can be simulated relatively quickly for use *in silico* studies.

To demonstrate the usefulness of the GRF model for the simulation of EM images of large sample volumes, some example datasets were simulated and are shown in Fig. 11. Fig. 11a shows a simulated image of a planar lamella with dimensions 400 × 400 × 150 nm containing 1500 apoferritin molecules and 48,927,000 atoms. The image has 4 *K* × 4 K pixels of size 1 Å. The time taken to produce the exit wave simulation scales with the number of atoms in the model and took ∼130 s to simulate using the GRF model and a full tilt series containing 90 images can be simulated in less than 2 h. Assuming a density of 0.94 g/cm^3^ this sample would contain more than 750 M water molecules; simulating a single image using a full atomic model for the ice would take more than a day and simulating a tilt series would be impractical. This serves to illustrate the convenience of being able to simulate the effect of the amorphous ice for large samples using the more computationally efficient GRF model.

Using the GRF model, the shape of the sample is produced by generating the random potential for each slice in the multislice calculation and then applying a mask such that the random potential is only applied to pixels which are expected to be amorphous ice. To perform this calculation efficiently, each voxel needs to be tested to determine whether it lies within the envelope defining the shape of the sample as it is rotated. For simple shapes, such as cuboids and cylinders, this can be calculated easily; however, samples with idealized shapes may not be appropriate for producing phantom datasets for testing data processing algorithms. Fig. 11b shows a simulated image of a non-ideal cylindrical sample containing 800 apoferritin molecules. This was produced by defining the deviation of the natural cylinder from the ideal cylinder using cubic splines to vary the offset from the cylinder axis and the radius as a function of distance along the cylinder axis. In this way, the GRF model can be used to produce complex sample geometries that mimic the properties of experimental cryo-EM samples.

## 4 Conclusions

The simulation of EM images of biological samples requires the use of accurate models of the amorphous ice in the sample. For small sample volumes, a physically realistic atom-based ice model can be utilised where individual atomic coordinates are used. However, for large sample volumes, the number of atoms in the physical model makes any form of molecular dynamics simulation computationally impractical. In these scenarios, computationally efficient models such as the GRF approach described here can be used with no measurable effect on the accuracy of the image simulations. As an example, a 400 × 400 × 150 nm planar lamella sample took ∼130 s to simulate on a Nvidia Quadro P4000 GPU, representing *a* > 400x speed up for large sample volumes compared to a physical model. Even for a smaller ice sample (645^3^ Å^3^), the speed up was 10x.

When simulating a tomographic tilt series, the positions of the water molecules remain fixed relative to one another as the sample is rotated in the atom-based model. In contrast, since the GRF model is generated on-the-fly for each image, the atomic potential of the amorphous ice will not correspond to water molecules with a fixed relative position as the sample is rotated. This could be seen as a limitation of the GRF model. However, in reality, the high-resolution structure of the amorphous ice changes as a result of exposure to the electron beam, with individual water molecules showing a movement of approximately 5 Å for every 25 e/Å^2^ [9,63]. For this reason, comparison of the statistical properties of the two ice models demonstrates that modelling the atomic potential of the amorphous ice as a GRF results in simulated images that are indistinguishable from those simulated from a physical atom-based model. In addition, tomographic reconstructions from the GRF model show comparable quality, as assessed by the FSC average, to reconstructions from the physical atom-based model. The GRF model has been implemented using the MULTEM simulation software for use in the Parakeet digital twin software [4], and the physical atom-based models can be obtained online from Zenodo [20].

## Declaration of Competing Interest

The authors declare the following financial interests/personal relationships which may be considered as potential competing interests:

Co-author is editor-in-chief of Ultramicroscopy - A.K.

## Data Availability

As stated in the manuscript, software is available on github and data on zenodo.

## Appendix Supplementary materials

Image, application 1
