# Supplementary material for: Computational models of amorphous ice for accurate simulation of cryo-EM images of biological samples
Source: Ultramicroscopy. 2024 Feb;256:None. doi: 10.1016/j.ultramic.2023.113882 (PMC10730944; doi:10.1016/j.ultramic.2023.113882)
Supplement: Supplementary file 1 [file mmc1.docx]

Computational models of amorphous ice for accurate simulation of cryo-EM images of biological samples: supplementary material

# S1. Mean and variance correction factor parameters

Owing to the details of the simulation method used in MULTEM, the mean and variance in the simulations depends on the pixel size and the moments of the Gaussian random field model need to be multiplied by dimensionless correction values to match the physical simulation. This is done by interpolating a correction factor using precomputed lookup tables given in Table S1.

| Pixel area (Å^2^) | Mean Correction | Variance Correction |
| --- | --- | --- |
| 0.01 | 0.9868463 | 0.9541554 |
| 0.04 | 0.9720215 | 0.8166216 |
| 0.09 | 0.9314166 | 0.6689699 |
| 0.16 | 0.8892865 | 0.5352830 |
| 0.25 | 0.8399629 | 0.4072256 |
| 0.36 | 0.7687288 | 0.2966287 |
| 0.49 | 0.6911600 | 0.2091032 |
| 0.64 | 0.6189148 | 0.1423872 |
| 0.81 | 0.5518357 | 0.0937952 |
| 1.00 | 0.4864729 | 0.0613248 |
| 1.21 | 0.4168598 | 0.0384087 |
| 1.44 | 0.3555671 | 0.0243265 |
| 1.69 | 0.2970996 | 0.0154021 |
| 1.96 | 0.2512393 | 0.0096818 |
| 2.25 | 0.2067110 | 0.0059035 |
| 2.56 | 0.1729837 | 0.0033408 |
| 2.89 | 0.1447699 | 0.0021511 |
| 3.24 | 0.0306664 | 0.0002175 |
| 3.61 | 0.0656295 | 0.0001382 |

**Table S1.** Correction factor parameters for the mean and variance calculations.

# S2. MD simulation protocol

Early simulations of apoferritin in water showed a significant exodus of water molecules from within the cavity of the apoferritin molecule into the bulk solvent, leaving a large vacuous ‘bubble’. For the waters within the cavity, hereafter referred to as the internal waters, it was established that there should be approximately 5700 internal water molecules to conserve the same density of water within the cavity as well as in the bulk solvent. Despite the number of molecules remaining constant in the chosen simulation ensembles, and the box volumes attributing the expected density, these bubbles continued to occur within the cavity, with approximately 1500 internal waters moving into the bulk solvent. An example of these bubbles can be found in Figure S1. After a significant amount of time spent on trial-and-error methods to determine the cause of the bubble formation, it was suggested that attempting to equilibrate a system of this size may be beyond the scope of the implemented barostat. The same effect was not observed when the apoferritin molecule was embedded in a smaller water box during initial testing.

With this critical information established, it was important to confirm that the chosen protocol for equilibrating the apoferritin simulations had been effective at prevention the escape of internal waters. It is important to note that this could not be done by visual inspection, given the RAM requirements to visualise such a system using a GUI are far beyond the scope of a typical desktop computer. The water molecules were tracked as they moved from the internal water region (an approximate sphere with a diameter of ~70 Å) to the bulk solvent. Some transportation was expected as part of system relaxation, particularly at higher temperatures where the protein structure fluctuated more, and the water molecules behaved in agreement with the expected dynamics of a liquid.


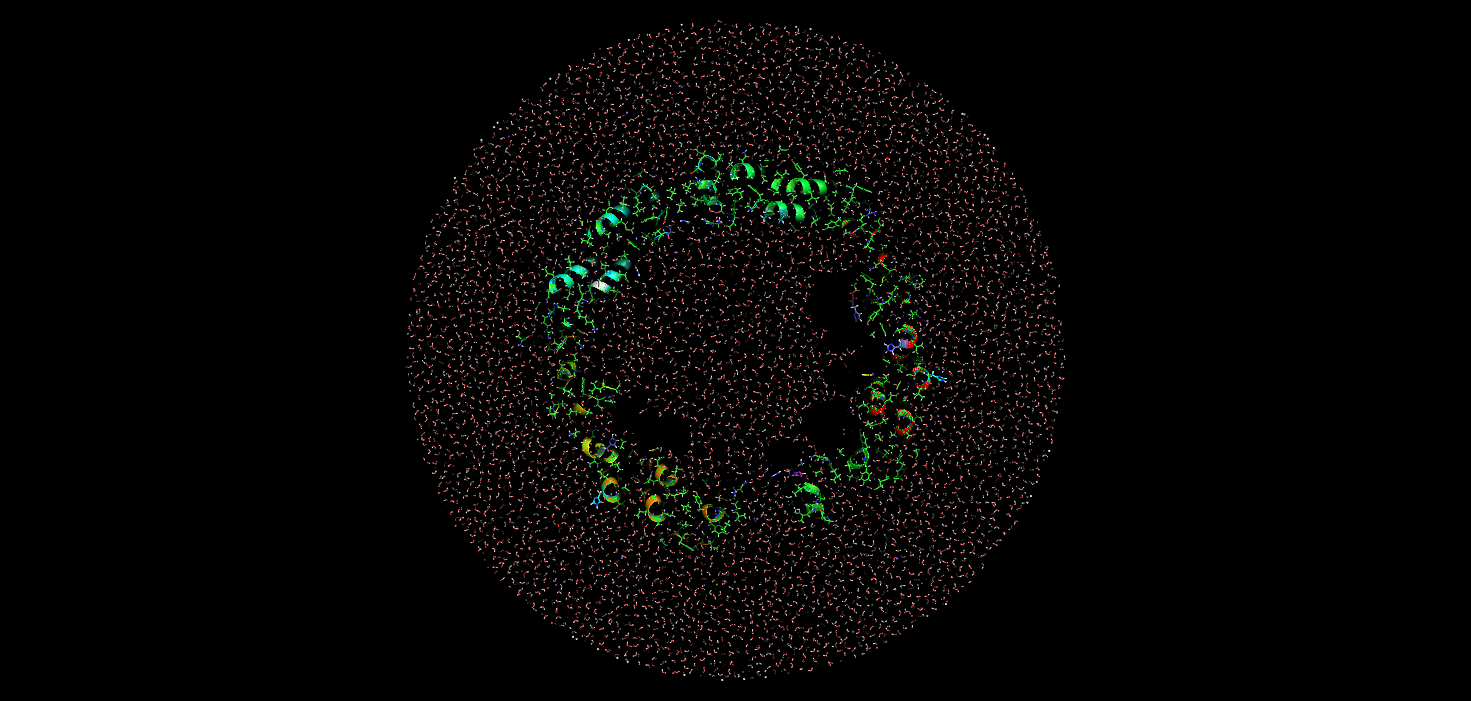


**Figure S1.** The vacuum holes left in the internal cavity of the apoferritin protein as a result of water molecule movement into the bulk solvent. Approximately 500 water molecules have transferred into the bulk solvent in the image shown.

The final protocol used for equilibrating the apoferritin system is presented in Table S2, as referenced in Section 2.2. Here, the individual simulation number and conditions are presented. By summing the simulation time from run 1 to 15, it is demonstrated that the total run time was 2.11 ns. Not included in this table is the extra restraint simulation used to restrain the positions of the apoferritin molecule back to the original crystal positions, thus preserving the molecular symmetry for analysis.

| Simulation Number | Timestep | Conditions | Simulation Time |
| --- | --- | --- | --- |
| 1, 50 K | 2 fs | NPT | 200 ps |
| 2, 100 K | 2 fs | NPT | 200 ps |
| 3, 150 K | 2 fs | NPT | 200 ps |
| 4, 220 K | 2 fs | NPT | 200 ps |
| 5, 250 K | 2 fs | NPT | 160 ps |
| 6, 280 K | 2 fs | NVT | 190 ps |
| 7, 298 K | 1 fs | NVT | 150 ps |
| 8, 298 K | 1fs | NVT | 150 ps |
| 9, 298 K | 1 fs | NVT | 150 ps |
| 10, 298 K | 1 fs | NVT | 150 ps |
| 11, 298 K | 1 fs | NVT | 150 ps |
| 12, 298 K | 1 fs | NVT | 150 ps |
| 13, 298 K | 1 fs | NPT | 10 ps |
| 14, 298 K | 1 fs | NVT | 25 ps |
| 15, 298 K | 1 fs | NPT | 25 ps |

**Table S2.** A list of the simulations run to fully equilibrate the apoferritin model. Each subsequent step was restarted from the final coordinates of the previous step

# S3. Ice model MD simulation results

The figure presented here confirm the expected behaviour of the molecular dynamics systems under the NPT ensemble. To confirm that the models were generating the expected structure of liquid water at 298K, the radial distribution function (RDF) for the 645^3^ Å^3^ system was calculated for the oxygen-oxygen distances, as shown in Figure S2. The first O-O peak at 2.8 Å reported in the RDF matches experimental findings (Hura *et al.*, 2003), however the TIP3P model fails to capture the subsequent solvation shells as distance increases. These findings are consistent with the behaviour of TIP3P water, as reported by Wang *et al.,* 2009. It should be noted that the RDF of the simulated water models may not exactly reflect the RDF of experiment LDA ice structures at low temperature due to the limitations of the water model used.


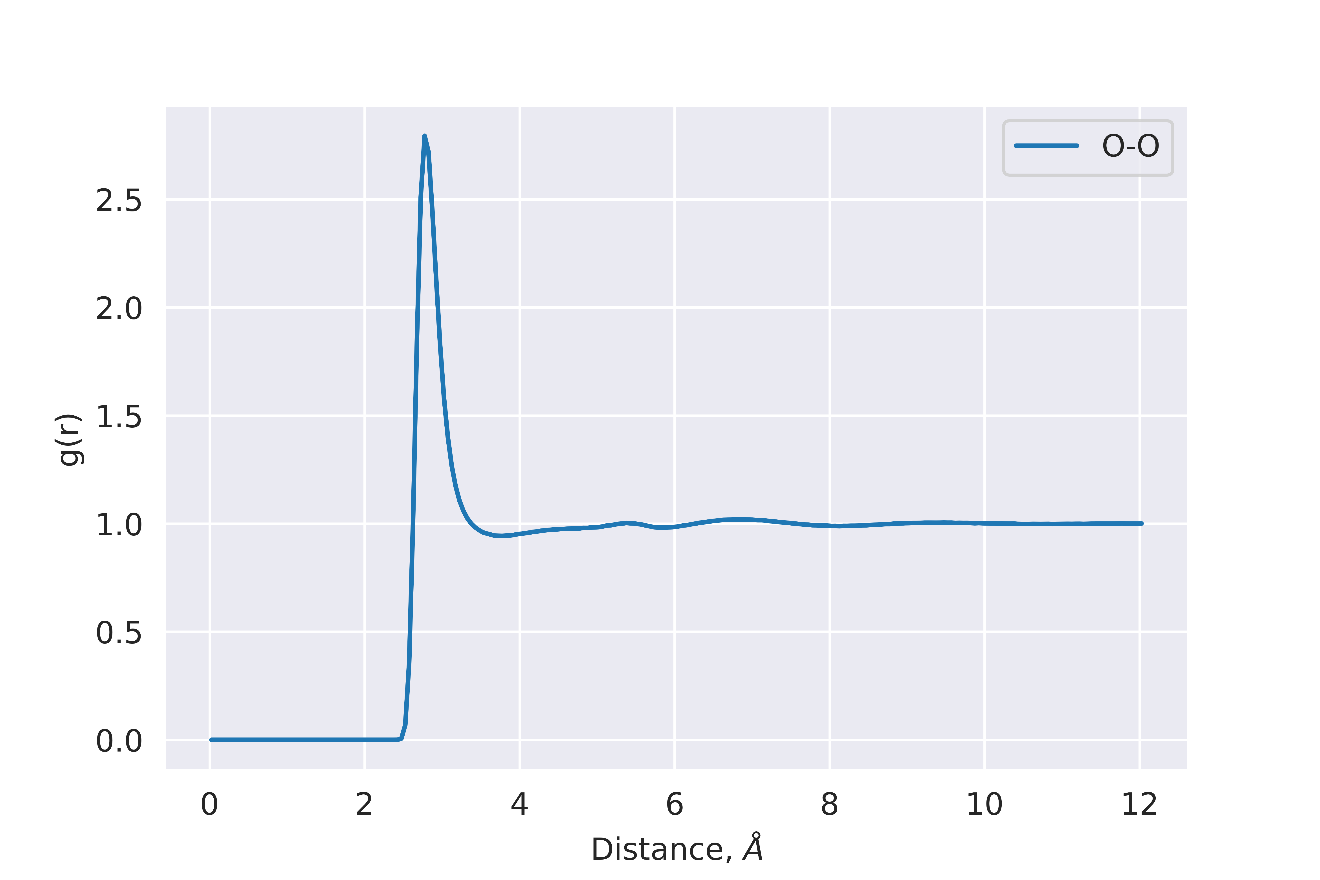


**Figure S2.** Radial distribution functions for the 645^3^ Å^3^ TIP3P water box at 298 K

# S4. EM image simulation parameters

The TEM images were simulated using the common set of parameters shown in Table S2. The pixel sizes varied for the different simulations as described in the text; typical image and simulation grid pixel sizes used in the simulations were between 0.1 Å to 1.0 Å. For the comparison with experimental data which was collected with a pixel size of 1.34 Å, the simulated images were produced with a pixel size of 0.335 Å and rebinned to 1.34 Å for the comparison.

| Parameter | Description | Value |
| --- | --- | --- |
| E | Energy | 300 keV |
| Δf | Defocus | 2.5 µm |
| C_3_ | Spherical aberration | 2.7 mm |
| C_c_ | Chromatic aberration | 2.7 mm |
| ΔI/I | Current spread | 0.33 ppm |
| ΔV/V | Voltage spread | 0.80 ppm |
| ΔE | Energy spread | 0.8 eV |
| θ_c_ | Source spread | 0.1 mrad |
| t_s_ | Simulation z-slice thickness | 5 Å |

**Table S3.** Parameters used in the TEM simulations, chosen to be consistent with parameters for a Thermo Fisher Scientific Titan Krios™ TEM (Thermo Fisher Scientific, 2020).

# References

Hura, G., Russo, D., Glaeser, R. M., Head-Gordon, T., Krack, M., Parrinello, M., (2003) “Water structure as a function of temperature from X-ray scattering experiments and *ab initio* molecular dynamics” Phys. Chem. Phys. 5, 1981-1991. <https://doi.org/10.1039/B301481A>

Thermo Fisher Scientific, (2020). Krios G4 Pre-Installation manual. Waltham, MA: Thermo Fisher Scientific.

Wang, H., Junghans, C., Kremer, K., (2009) “Comparative atomistic and coarse-grained study of water: What do we lose by coarse-graining?” *Eur. Phys. J. E.* 28, 221-229. <https://doi.org/10.1140/epje/i2008-10413-5>
